# Divergent mitochondrial and fibrotic signatures in atrial fibrillation: insights from organoids and GEO data

**DOI:** 10.1186/s12933-026-03291-0

**Published:** 2026-07-11

**Authors:** Jihee Won, Hyocheol Jung, Jin Chul Kim, Ji-Eun Jeong, Young Il Park, Biaggio Uricoli, Sanghee Lee, Young Hoon Son, Gun-Jae Jeong

**Affiliations:** 1 Coulter Department of Biomedical Engineering, Georgia Institute of Technology & Emory University School of Medicine, Atlanta, GA 30322 USA; 2 Center for Specialty Chemicals, Division of Specialty and Bio-based Chemical Technology, Korea Research Institute of Chemical Technology, Ulsan, 44412 Republic of Korea; 3 School of Nanomedical Engineering, Korea National University of Transportation, Chungju, 27469 Republic of Korea; 4 Institute of Cell and Tissue Engineering, College of Medicine, The Catholic University of Korea, Seoul, 06591 Republic of Korea

**Keywords:** Atrial fibrillation, Organoids, Angiotensin II, Rapid pacing, Metabolic remodeling, Mitochondrial dysfunction, GEO transcriptomics

## Abstract

**Background:**

Atrial fibrillation (AF) remains a major clinical burden because structural and metabolic remodeling, particularly mitochondrial dysfunction and fibrosis, often persists beyond rhythm control. Human-relevant models that disentangle these axes and connect them to patient data are limited.

**Methods:**

Human atrial organoids were exposed to two AF-relevant stressors: rapid pacing (FP) and angiotensin II (AngII). We assessed mitochondrial and fibrotic remodeling by transcript/protein analyses, imaging, and contractility readouts, and benchmarked organoid responses against four public AF transcriptomic datasets (GEO: GSE128188, GSE138252, GSE222793, GSE239321). Statistical analyses used predefined contrasts versus matched controls with multiple-testing correction.

**Results:**

FP predominantly suppressed mitochondrial biogenesis/oxidative programs and increased stress-response features, resembling signatures observed in AF with heart failure (e.g., downregulation of mitochondrial regulators and altered cytochrome c patterns). AngII elicited stronger profibrotic signaling with a mixed mitochondrial response, mirroring profiles more typical of AF without heart failure. Both stressors impaired organoid contractility. Protein and immunostaining corroborated mitochondria–fibrosis crosstalk, with changes in PGC1α and cytochrome c alongside increased extracellular matrix deposition and myofibroblast markers (COL1A1, αSMA), consistent with stimulus-specific but partially overlapping remodeling trajectories.

**Conclusion:**

Distinct AF-relevant stressors drive divergent mitochondrial and fibrotic remodeling in human atrial organoids that recapitulate key features of patient datasets. These organoid models provide a translational platform to prioritize mitochondria-targeted and antifibrotic interventions and to inform biomarker development for residual dysfunction in AF.

**Supplementary Information:**

The online version contains supplementary material available at https://doi.org/10.1186/s12933-026-03291-0.

## Research insights

**What is currently known about this topic?**

- AF persistence is driven by mitochondrial dysfunction and fibrotic remodeling.
- Cardiometabolic conditions worsen AF by creating a profibrotic atrial environment.
- Existing 2D and animal models fail to fully reflect complex human atrial tissue changes.

**What is the key research question?**

- How do distinct AF stressors drive metabolic and fibrotic remodeling in human atrial organoids?

**What is new?**

- Rapid pacing suppressed mitochondria, matching clinical data of AF with heart failure.
- Angiotensin II drove strong fibrosis, resembling profiles of AF without heart failure.
- Fibrosis is a more universally conserved transcriptional AF hallmark than metabolic shifts.

**How might this study influence clinical practice?**

- Human organoids provide a platform to screen metabolic therapies for structural AF remodeling.

## Background

Atrial fibrillation (AF) is the most common sustained cardiac arrhythmia, affecting more than 52 million people worldwide and greatly increasing the risk of stroke, heart failure, and mortality [1, 2]. Despite major advances in arrhythmia management, the prevalence of AF continues to rise, underscoring the need for deeper mechanistic insight [3]. Most research to date has emphasized the electrophysiological basis of AF initiation, focusing on ion channel remodeling, calcium handling abnormalities, and reentrant conduction circuits [4–6]. While these insights have shaped rhythm-control strategies, they do not adequately explain the progressive tissue remodeling that promotes AF persistence, recurrence, and long-term functional decline [7, 8]. Importantly, the increasing clinical burden of AF is closely intertwined with the global rise in cardiometabolic diseases, such as diabetes and metabolic syndrome, which create a pro-oxidative and profibrotic environment in the heart tissue [9–11].

Structural and metabolic remodeling of atrial tissue is now recognized as a critical driver of AF pathogenesis in these clinical contexts [12, 13]. Two features are particularly important: mitochondrial dysfunction and fibrosis [14–16]. Healthy mitochondria are crucial for sustaining the high energy demands of atrial cardiomyocytes and for regulating oxidative stress, calcium balance, and cell survival [14]. In AF, impaired oxidative phosphorylation, reduced biogenesis, and disrupted mitochondrial dynamics have been reported, although whether these changes are causal or secondary to disease progression remains uncertain [17, 18]. At the same time, AF is driven by fibroblast activation, TGF-β signaling, and extracellular matrix accumulation [19, 20]. This process reduces tissue compliance, slows electrical conduction, and creates a substrate that promotes reentry, ultimately contributing to chronic remodeling that persists even after sinus rhythm is restored [21, 22].

Importantly, mitochondrial dysfunction and fibrosis are not independent phenomena but form a reinforcing loop. Excessive reactive oxygen species (ROS) generated by dysfunctional mitochondria activate pro-fibrotic pathways, including TGF-β/SMAD signaling, while mitochondrial damage-associated molecular patterns further stimulate fibroblast activation and extracellular matrix deposition [23–25]. In turn, fibrotic remodeling stiffens atrial tissue, alters mechano-electric coupling, and impairs oxygen and substrate delivery, which exacerbates mitochondrial stress and bioenergetic decline [26–28]. This bidirectional crosstalk establishes devastating cycle that stabilizes the AF substrate and accelerates disease progression.

However, existing models offer limited insight into these remodeling mechanisms. Animal models replicate arrhythmia initiation but differ from human atrial physiology and disease progression [29, 30]. Two-dimensional cell cultures, although experimentally tractable, lack the multicellular architecture and paracrine interactions that underline fibrosis and metabolic adaptation [31, 32]. Moreover, most experimental systems are designed to induce arrhythmia rather than to interrogate the remodeling that characterizes AF survivors, who often remain symptomatic due to structural and energetic compromise.

Organoid platforms offer an opportunity to bridge these gaps. Unlike single-lineage cardiomyocyte cultures, organoids incorporate multiple cardiac cell types, enabling paracrine signaling, matrix deposition, and tissue-level interactions resembling the native atrial environment [33, 34]. This multicellular complexity is particularly valuable for studying fibrosis and mitochondrial remodeling, which emerge from crosstalk between myocytes, fibroblasts, and supporting cells [35–37]. While organoids do not fully reproduce the structure of human atria, they provide an intermediate system between in vitro and in vivo models, with the added advantage of alignment to patient-derived datasets.

In this study, we established two human atrial organoid models of AF remodeling. One model was subjected to rapid pacing (FP), mimicking electrophysiological stress, while the other was exposed to angiotensin II (AngII), representing neurohormonal activation and key mediator of fibrotic remodeling in cardiometabolic diseases such as diabetic cardiomyopathy. We complemented these experimental models with analysis of four publicly available human AF transcriptomic datasets (GSE128188, GSE138252, GSE222793, and GSE239321) to identify conserved and divergent remodeling signatures. By integrating organoid phenotyping with patient-derived data, we aimed to define how different triggers influence mitochondrial and fibrotic remodeling and to evaluate the utility of atrial organoids as a translational platform for investigating post-AF tissue remodeling and identifying potential therapeutic targets for cardiometabolic associated arrhythmias.

## Methods

### Data compilation and processing

We queried the Gene Expression Omnibus (GEO) repository using the following terms: “Atrial Fibrillation” [All Fields] AND “Homo sapiens” [porgn: txid9606] AND “Expression profiling by high throughput sequencing” [Filter]. Four RNA-seq datasets: GSE128188, GSE138252, GSE222793, and GSE239321 were selected for further processing (Table 1). Only samples of interest were included, excluding cells subjected to any treatment. Dataset-specific inclusion and exclusion criteria are described in Table 1. Raw count matrices were downloaded from GEO and processed as described in “Differential expression and pathway enrichment analysis” section.

**Table 1:** Summary of GEO transcriptomic datasets used in this study

GEO ID	Sample type	Biological model	Comparison	Inclusion/Exclusion criteria	Role in study	N
GSE128188	Human left atrial appendage tissue	In vivo AF (chronic, permanent)	AF vs SR	LAA samples only; RAA excluded; permanent AF cases vs SR controls	Core in vivo AF transcriptional signature	5/5
GSE138252	Human left atrial cardiomyocytes (cardiomyocyte-specific nuclear RNA)	AF with concurrent Heart Failure	AF + HF vs NF	LA cardiomyocytes only; LV, RV, RA, HF-only, and ploidy variant samples excluded	Cardiomyocyte-specific remodeling under AF + HF	5/5
GSE222793	iPSC-derived atrial-like cardiomyocytes	Metabolic spectrum of arrhythmogenic cardiomyopathy	Adipogenic vs Basal medium	All clones included; basal and adipogenic conditions only; treatment-naive cells at matched passage	Metabolic stress induction of AF-like transcriptional program	10/10
GSE239321	Primary human cardiac fibroblasts	Atrial vs ventricular fibroblast identity	Atrial vs Ventricular FB	Atrial and ventricular fibroblasts only; 2 donors, 2 replicates each; no AF vs SR contrast	Chamber-specific fibrotic reference; atrial fibroblast profibrotic identity	4/4

N = number of samples per group (Group A / Group B). LAA = left atrial appendage; FB = fibroblasts; iPSC = induced pluripotent stem cells; SR = sinus rhythm; AF = atrial fibrillation; HF = heart failure. All datasets used Illumina RNA-seq platforms

These four datasets represent complementary biological contexts of AF-related remodeling and are not equivalent replicates of a single comparison. Cross-dataset integration was performed at the pathway level using gene set enrichment analysis (GSEA) followed by Fisher combined probability meta-analysis, which is robust to biological heterogeneity across models

### Culture of human-induced pluripotent stem cells (hiPSCs)

Human-induced pluripotent stem cells (hiPSCs; STEMCELL TECHNOLOGIES) were cultured according to established protocols for human pluripotent stem cell maintenance [38–41]. Cryopreserved hiPSCs were thawed and seeded as small aggregates onto Matrigel-coated 6-well plates in mTeSR™ medium (STEMCELL TECHNOLOGIES) supplemented with 10 µM Y-27632 (ROCK inhibitor, Sigma Aldrich). After 24 h, the medium was replaced with mTeSR™ medium without Y-27632. Cells were maintained for 5–7 days with daily medium changes until compact colonies reached a size suitable for passaging or experimental use.

### Generation of a cardiac organoid composed of atrial cardiomyocytes

Cardiac organoids were generated as previously described [42], with minor modifications. The atrial identity and characterization of this organoid platform have been described previously [42]. Four days before organoid induction, hiPSC colonies were washed with calcium- and magnesium-free phosphate buffered saline (PBS) and dissociated with 500 µL Accutase (Gibco) per well in a six-well plate. After 8–10 min at 37 °C, cells were gently triturated to obtain single-cell suspensions, transferred to a 50 mL conical tube. Residual hiPSCs remaining in the wells were collected by rinsing each well with 1.5 mL mTeSR™ medium supplemented with 10 µM Y-27632, and the rinse was combined with the initial suspension. Cells were pelleted by centrifugation at 300×*g* for 5 min, resuspended in mTeSR™ with Y-27632, and seeded into low-attachment U-bottom 96-well plates at 5000 cells per well to allow aggregate formation. After 48 h, 20 µL Matrigel (Corning) droplets were dispensed into fresh low-attachment U-bottom 96-well plates, and individual hiPSC aggregates were carefully transferred into the droplets for embedding. Droplets were polymerized at 37 °C for 50–60 min before adding 100 µL mTeSR™ medium without Y-27632. Following an additional 48 h, cultures were sequentially transitioned to 200 µL of atrial cardiomyocyte differentiation media A, B, and C (STEMCELL TECHNOLOGIES), with media A and B maintained for 48 h each. For medium C, cultures were exposed for 48 h, then the medium was replaced with fresh medium C and maintained for another 48 h (total 96 h) before transition to cardiomyocyte maintenance medium (STEMCELL TECHNOLOGIES), which was changed every 48 h thereafter. After 15 days of differentiation, three independent cases were prepared for each experimental group (control, FP, or AngII treatment), with each case comprising 26 Matrigel droplets containing one cardiac organoid each. Droplets were pooled per case, resuspended in 400 µL Matrigel following medium removal, and distributed into six-well plates. Gels were solidified at 37 °C in 5% CO₂ for 1 h, and organoids were subsequently maintained in cardiomyocyte maintenance medium under their respective treatment conditions.

### Induction of atrial fibrillation in cardiac organoids using fast pacing and angiotensin II treatment

Re-embedded cardiac organoids maintained in bulk Matrigel were subsequently subjected to atrial fibrillation-like stimulation using either fast pacing (FP) or AngII treatment. Atrial fibrillation–like conditions were induced by applying electrical pacing at 3 Hz for 24 h using biphasic 5 ms pulses at 10 V, delivered by a C-PACE EP culture pacer (IonOptix, Dublin, Ireland). In parallel, organoids were treated with 1 μM AngII for 24 h. Organoids were subsequently harvested for downstream analyses.

### Harvest of organoids for analysis

Cardiac organoids embedded in Matrigel were collected from each well of a six-well plate into a 50 mL conical tube. After removal of the supernatant, organoids were incubated with Cell Recovery Solution (Corning) at 4 °C for 1 h with occasional gentle mixing to dissolve the Matrigel. The solution was discarded, and organoids were washed 2–3 times with cold PBS. Treatment with Cell Recovery Solution and PBS washing were repeated as necessary until the Matrigel was completely removed. The harvested organoids were subsequently processed for histology, qRT-PCR, and western blot analysis.

### Immunofluorescence staining of cardiac organoids

Three organoids from each condition were fixed in 4% formaldehyde for 10 min at room temperature, followed by overnight fixation at 4 °C. Fixed organoids were washed twice with PBS and cryoprotected by sequential incubation in 15% and 30% sucrose solutions at 4 °C for 6 h to overnight, until the organoids sank to the bottom. Organoids were then embedded in OCT compound (Sakura), frozen in liquid nitrogen within an insulated chamber, and stored at  − 80 °C overnight. OCT blocks were sectioned using a cryostat (Leica Microsystems). Sections were air-dried for 30 min at roomtemperature, rinsed twice with PBS (3 min each), and washed three times with PBST (PBS containing 0.05% Tween-20; 5 min each). Permeabilization was performed with Triton X-100 (Sigma Aldrich) under the following conditions: 0.1% for 5 min (COL1A1) or 10 min (SMA), 0.2% for 10 min (Cytochrome C), and 0.3% for 10 min (PGC1a and MYH7). Sections were blocked with 4% bovine serum albumin (BSA; Sigma Aldrich) in PBST for 1 h at room temperature, followed by five 10-min washes in PBST. Primary antibodies were applied overnight at 4 °C in PBST using the following dilutions: PGC1a (Abcam, ab191838, 1:500), Cytochrome C (Abcam, ab13575, 1 µg/mL), COL1A1 (Santa Cruz, sc-293182, 1:50), SMA (Santa Cruz, sc-53015, 1:50), and MYH7 (Santa Cruz, sc-53089, 1:50). Sections were washed five times in PBST (10 min each) and incubated with Alexa Fluor 488-conjugated secondary antibodies (Invitrogen) for 1 h at room temperature. After five additional washes in PBST (5 min each), sections were incubated with Alexa Fluor 594 phalloidin (Invitrogen) for 30 min at room temperature. Following another five PBST washes (10 min each), sections were counterstained with DAPI (Invitrogen) for 10 min at room temperature and rinsed three times in PBS (5 min each). The residual liquid was removed, and one drop of ProLong™ Glass Antifade Mountant (Invitrogen) was applied to each specimen. Coverslips were carefully placed onto the mountant to avoid trapping air bubbles. Mounted samples were kept on a flat, dry surface and allowed to cure overnight at room temperature, protected from light. Immunofluorescence images were acquired using an AxioVision 7 imaging system (Zeiss).

### Differential expression and pathway enrichment analysis

All four GEO datasets (GSE128188, GSE138252, GSE222793, and GSE239321) were RNA-seq datasets processed using the limma-voom pipeline implemented in R (Bioconductor). Raw count matrices for each dataset were downloaded from GEO and low-expression genes were filtered prior to analysis, retaining genes with counts per million (CPM) > 1 in at least the number of samples corresponding to the smaller group in each comparison. Library sizes were normalized using the trimmed mean of M-values (TMM) method via the edgeR package to account for compositional differences between samples. Normalized counts were transformed using the voom function, which estimates the mean–variance relationship of log-counts and assigns precision weights to each observation, rendering the data suitable for linear modeling. A linear model was fitted to each gene using the lmFit function, and differential expression was assessed using empirical Bayes moderation of standard errors via the eBayes function, which stabilizes variance estimates across genes, particularly for datasets with small sample sizes. Dataset-specific group contrasts were defined as follows: in GSE128188, atrial fibrillation (AF) versus sinus rhythm (SR) in left atrial appendage tissue; in GSE138252, AF with concurrent heart failure (AF + HF) versus non-failing controls in atrial cardiomyocytes; in GSE222793, adipogenic medium versus basal condition in iPSC-derived atrial-like cardiomyocytes; and in GSE239321, atrial versus ventricular fibroblast identity. Genes with an absolute log₂-fold change greater than 1 and a Benjamini–Hochberg-adjusted *p* value less than 0.05 were classified as significantly differentially expressed genes (DEGs). Results were visualized as volcano plots displaying − log₁₀(adjusted *p* value) against log₂FC, with upregulated genes highlighted in blue and downregulated genes in red.

To assess pathway-level transcriptional changes, gene set enrichment analysis (GSEA) was performed on a per-dataset basis using the fgsea package in R. Genes were pre-ranked by the moderated t-statistic derived from the limma-voom output, and enrichment was calculated against the Hallmark and Gene Ontology (GO) gene set collections from MSigDB (v2023.1). A minimum gene set size of 15 and maximum of 500 was applied. Statistical significance was assessed using 1000 permutations, and gene sets with a Benjamini–Hochberg-adjusted *p* value below 0.05 and a normalized enrichment score (NES) with absolute value greater than 1 were considered significantly enriched. Cross-dataset pathway convergence was evaluated by Fisher’s combined probability method applied to the per-dataset adjusted *p* values for each gene set, providing a meta-analytic summary of consistently enriched biological processes across the four AF transcriptomic contexts. Functional enrichment analysis for overrepresentation of Gene Ontology (GO) biological process, molecular function, and cellular component terms, as well as Kyoto Encyclopedia of Genes and Genomes (KEGG) pathways, was additionally performed on the DEG lists using the clusterProfiler package in R, with a background defined as all expressed genes passing the CPM filter. All analyses were conducted in R version 4.3.1.

### Directional concordance analysis between organoid and GEO datasets

To quantitatively compare experimentally measured organoid responses with publicly available transcriptomic datasets, we performed a targeted directional concordance analysis. For each AF induction model (FP and AngII), genes experimentally measured by qPCR were matched to the corresponding genes in each GEO dataset. Directional concordance was defined as the percentage of shared genes exhibiting the same direction of regulation (positive or negative log₂ fold change) between the organoid data and the GEO dataset. Separate analyses were performed for the mitochondrial gene panel (22 genes) and the fibrosis-related gene panel (9 genes). Because organoid genome-wide RNA sequencing was not performed, concordance analysis was limited to experimentally validated genes rather than whole-transcriptome comparisons.

### Gene expression analysis of cardiomyocytes

To extract total RNA from the organoids using TRI reagent Invitrogen, Carlsbad, CA), and reverse transcription was performed using Superscript II and oligo(dT) primers (Invitrogen). Primer sequences are listed in Supplementary Table 1. qRT-PCR analysis was performed using StepOnePlus (Applied Biosystems, Foster City, CA). For each sample, 20 ng total cDNA were subjected to qRT-PCR in duplicate according to the manufacturer’s protocol. The analysis involved calculating 2-ΔCt values from the Ct values, using GAPDH as the housekeeping gene for normalization and comparing the expression levels to those of the control group.

### Western blot of cardiomyocytes

Samples were homogenized in RIPA buffer supplemented with PhosSTOP Easypack phosphatase inhibitors and cOmplete Mini Easypack protease inhibitor tablets. Equal amounts of protein were separated by sodium dodecyl sulfate–polyacrylamide gel electrophoresis (SDS-PAGE) and transferred onto nitrocellulose membranes. Membranes were probed with the following primary antibodies: PGC1α (ab191838), cytochrome C (ab13575) from Abcam; Col1a1 (sc-271249), SMA (sc-53142) from Santa Cruz Biotechmology; and GAPDH (G9545) from Sigma-Aldrich.

### Video analysis using musclemotion software

To assess the contractile behavior of cardiac organoids, live video capture was performed using a Zeiss LSM 780 microscope operating in transmitted light mode. Videos were recorded at 60 frames per second over a continuous 30-s duration. These videos were then converted to.avi format utilizing ImageJ software. Organoids were selected for video analysis based on pre-defined morphological criteria: intact spheroid structure, absence of necrotic regions, and confirmed spontaneous contractile activity. All qualifying organoids were included; 3–5 per biological replicate were recorded. The contraction frequency of the organoids was quantified through manual review of the footage. Comprehensive contraction profiles were generated using MUSCLEMOTION, an open-source tool, following the operational protocols recommended by the tool’s developers, to accurately gauge the contractile dynamics of the organoids.

### Statistical analysis

All statistical analyses were performed using Prism version 10 (GraphPad Software, San Diego, CA). Data were assessed for normality using the Shapiro–Wilk test, and comparisons between groups were conducted using one-way ANOVA followed by Tukey’s post hoc test for multiple comparisons. For non-normally distributed data, the Wilcoxon matched-pairs signed-rank test was applied. A *p* value < 0.05 was considered statistically significant. Data are presented as mean ± SEM.

## Results

### Human AF datasets reflect complementary clinical and experimental models

**Fig. 1:**
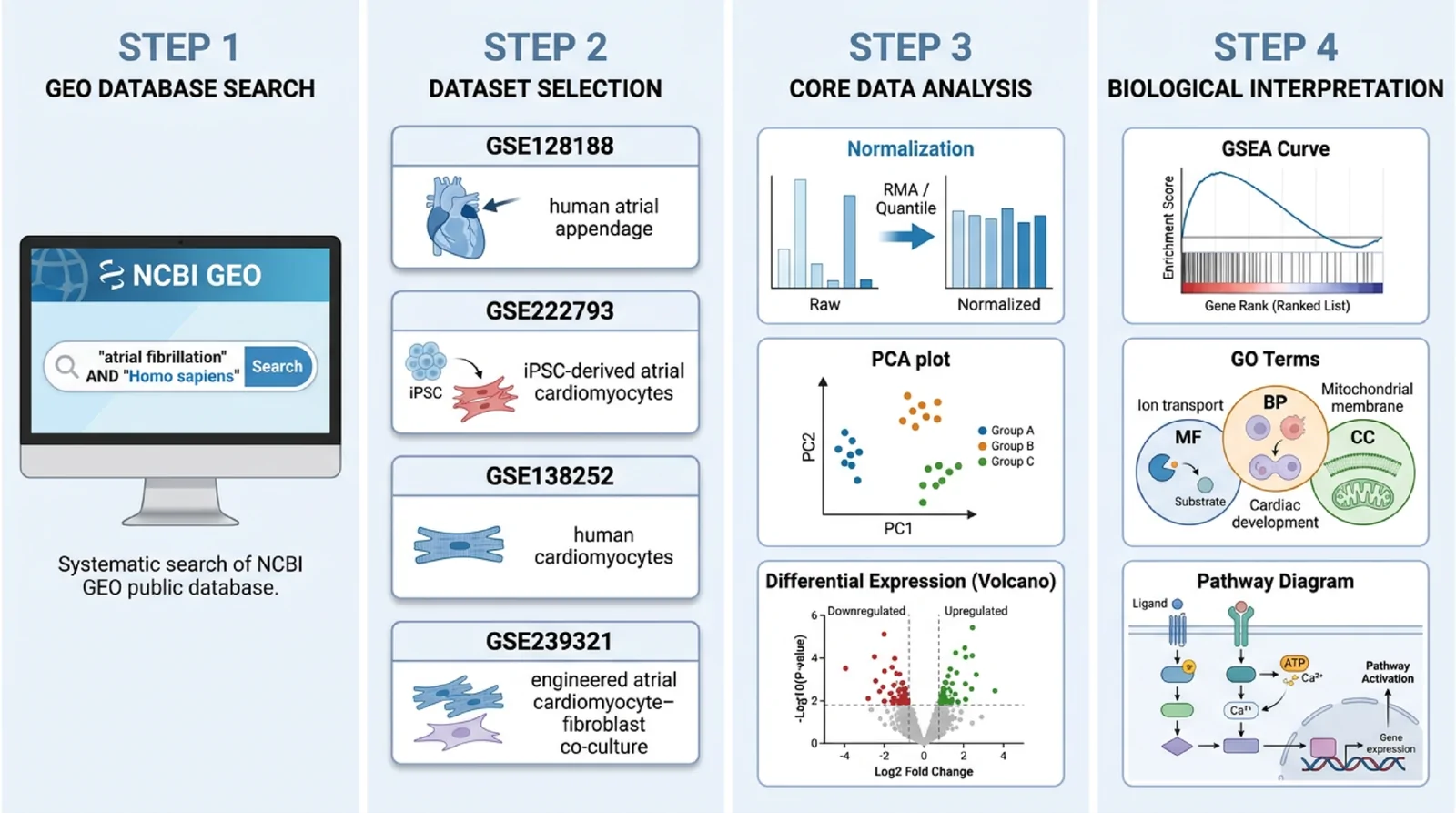
Schematic overview of selection and processing of human AF datasets from the GEO database. Step 1: Relevant human transcriptomic datasets were identified through systematic searches of the NCBI Gene Expression Omnibus (GEO) database using AF-related keywords. Step 2: Four complementary datasets representing distinct biological contexts were selected: GSE128188 (human atrial appendage tissue), GSE222793 (iPSC derived atrial cardiomyocytes), GSE138252 (human cardiomyocytes), and GSE239321 (engineered atrial cardiomyocyte fibroblast coculture system). Step 3: Data preprocessing and analysis included normalization (e.g., RMA/quantile normalization), principal component analysis (PCA) to assess sample clustering, and differential expression analysis to identify significantly altered genes between conditions. Step 4: Functional interpretation of differentially expressed genes was performed using gene set enrichment analysis (GSEA), Gene Ontology (GO) annotation (molecular function, biological process, and cellular component), and pathway analysis to identify key biological mechanisms associated with AF

To capture conserved molecular signatures across the AF spectrum, we compiled four GEO transcriptomic datasets representing complementary biological contexts (Fig. 1). GSE128188 comprises RNA-sequencing data from paired left atrial appendages of chronic AF patients, directly reflecting in vivo atrial remodeling. GSE138252 contains cardiomyocyte-specific nuclear expression profiles from AF with concurrent heart failure, representing advanced bioenergetic and structural compromise. GSE222793 employs iPSC-derived atrial-like cardiomyocytes under adipogenic medium to model the cardiometabolic predisposition associated with obesity- and diabetes-related AF susceptibility. GSE239321 profiles atrial versus ventricular fibroblast identity, establishing the intrinsic fibrotic substrate of the atrial myocardium. Together, these datasets span a progressive AF spectrum from structural predisposition to established disease with end-organ failure. Differential expression analysis was performed using the limma-voom pipeline (R/Bioconductor), and pathway enrichment was assessed using the fgsea package, with cross-dataset convergence evaluated by Fisher's combined probability meta-analysis, providing rigorous gene signatures for downstream pathway enrichment and benchmarking of organoid data.

### Transcriptomic analysis identifies conserved fibrotic and context-dependent mitochondrial signatures across the AF spectrum

**Fig. 2:**
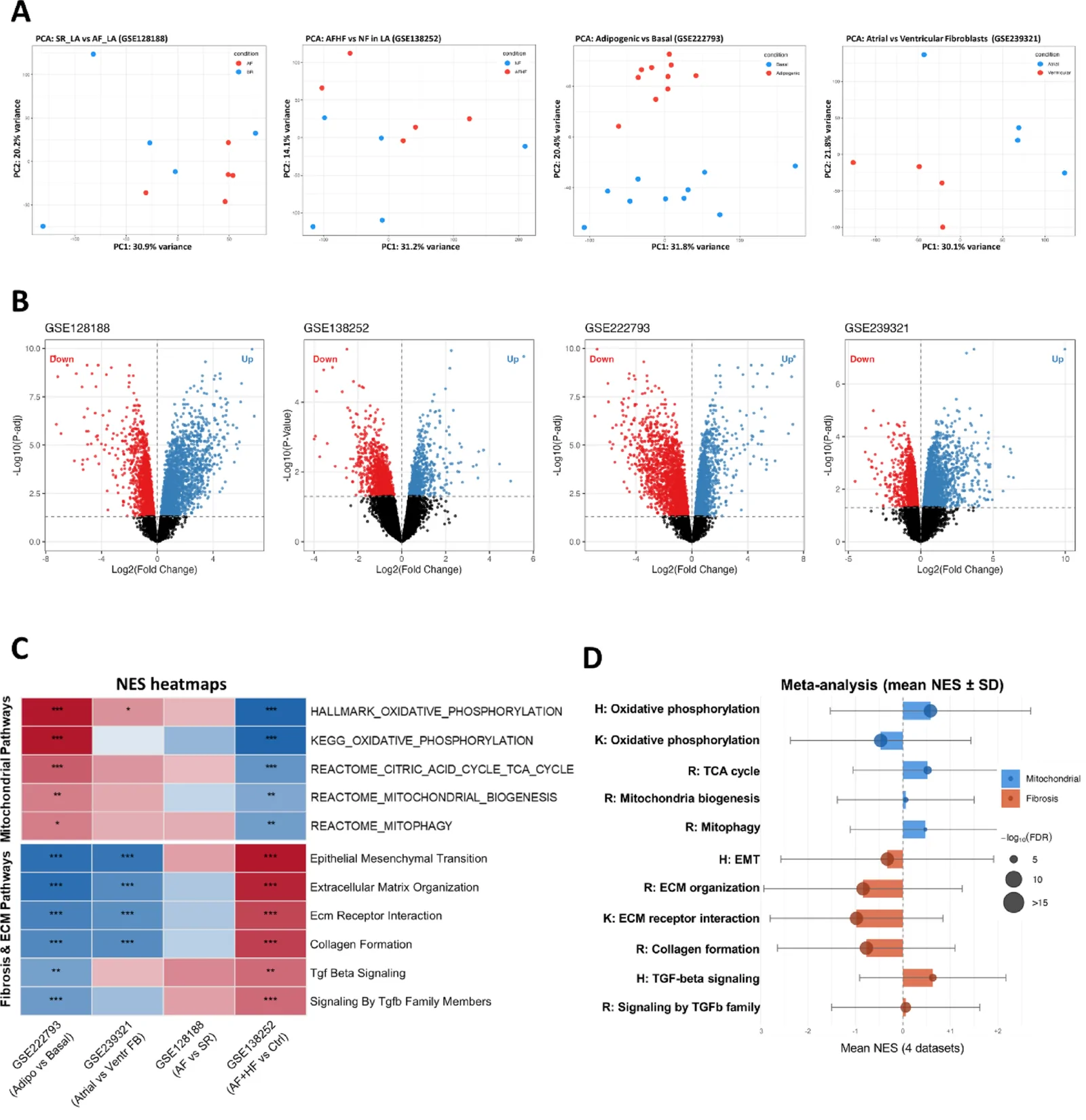
Cross-dataset transcriptomic analysis across the atrial fibrillation spectrum. **A** Principal component analysis (PCA) of normalized gene expression across four GEO datasets representing complementary AF biological contexts. Each point represents an individual sample colored by experimental group. GSE128188: atrial fibrillation (AF, red) vs. sinus rhythm (SR, blue) in left atrial appendage; GSE138252: AF with heart failure (AF + HF, red) vs. non-failing controls (NF, blue); GSE222793: adipogenic medium (red) vs. basal medium (blue) in iPSC-derived atrial cardiomyocytes; GSE239321: atrial fibroblasts (red) vs. ventricular fibroblasts (blue). Clear separation between groups confirms dataset quality and biological signal. **B** Volcano plots displaying differentially expressed genes across four GEO datasets spanning the AF spectrum. The x-axis shows log₂ fold change and the y-axis shows − log₁₀ (adjusted *p* value). Blue: upregulated; red: downregulated (|log₂FC|> 1, BH-adjusted *p* < *0.05*). **C** Normalized enrichment score (NES) heatmap of mitochondrial (top) and fibrosis-associated (bottom) pathways across four GEO datasets (GSE222793, GSE239321, GSE128188, and GSE138252), ordered from cardiometabolic predisposition to advanced AF with heart failure, representing a progressive AF spectrum. Pathways include oxidative phosphorylation, TCA cycle, mitochondrial biogenesis, and mitophagy (top), and epithelial-mesenchymal transition, ECM organization, collagen formation, and TGF-β signaling (bottom). Blue: negative enrichment; red: positive enrichment in the case condition relative to control. Significance: * *padj* < *0.05*, ** *padj* < *0.01*, *** *padj* < *0.001*. **D** Meta-analysis of pathway enrichment showing mean NES ± SD across all four datasets for mitochondrial (blue) and fibrosis-associated (orange) pathways. Point size reflects − log₁₀ (Fisher combined FDR), where larger points indicate greater cross-dataset statistical significance. The dashed vertical line marks NES = 0. Mitochondrial pathways showed context-dependent enrichment across the AF spectrum, while fibrosis-associated pathways demonstrated consistently high cross-dataset significance (Fisher combined FDR < 10⁻^1^⁹). All data are presented as mean ± SD. *p < *0.05*, **p < *0.01*, ***p < *0.001*

Despite differences in sample origin, transcriptomic profiling across four human AF datasets (GSE128188, GSE138252, GSE222793, and GSE239321) revealed extensive transcriptional remodeling. PCA plots (Fig. 2A) show partial separation between conditions across all four datasets. Volcano plots (Fig. 2B) illustrate gene expression changes in each dataset, with each comparison exhibiting hundreds of differentially expressed genes and clear separation between upregulated and downregulated transcripts. NES heatmaps show distinct pathway enrichment patterns across the four datasets (Fig. 2C). Mitochondrial pathway gene sets, including oxidative phosphorylation, TCA cycle, mitochondrial biogenesis, and mitophagy, showed strong suppression in GSE138252 but variable enrichment in the remaining datasets, reflecting distinct metabolic states across the AF spectrum. Fibrosis-associated gene sets including epithelial-mesenchymal transition, ECM organization, collagen formation, and TGF-β signaling showed strong but directionally variable enrichment, with upregulation in GSE138252 and context-dependent patterns in the remaining datasets. Fisher combined probability meta-analysis confirmed that fibrosis-associated pathways achieved the highest cross-dataset statistical significance, while mitochondrial pathway enrichment remained stage-dependent (Fig. 2D). These results suggest that both mitochondrial dysfunction and fibrotic remodeling are transcriptionally prominent across the AF spectrum, with direction and magnitude reflecting the distinct biological context of each dataset.

### Electrical and hormonal stress impair contractile function in atrial organoids

**Fig. 3:**
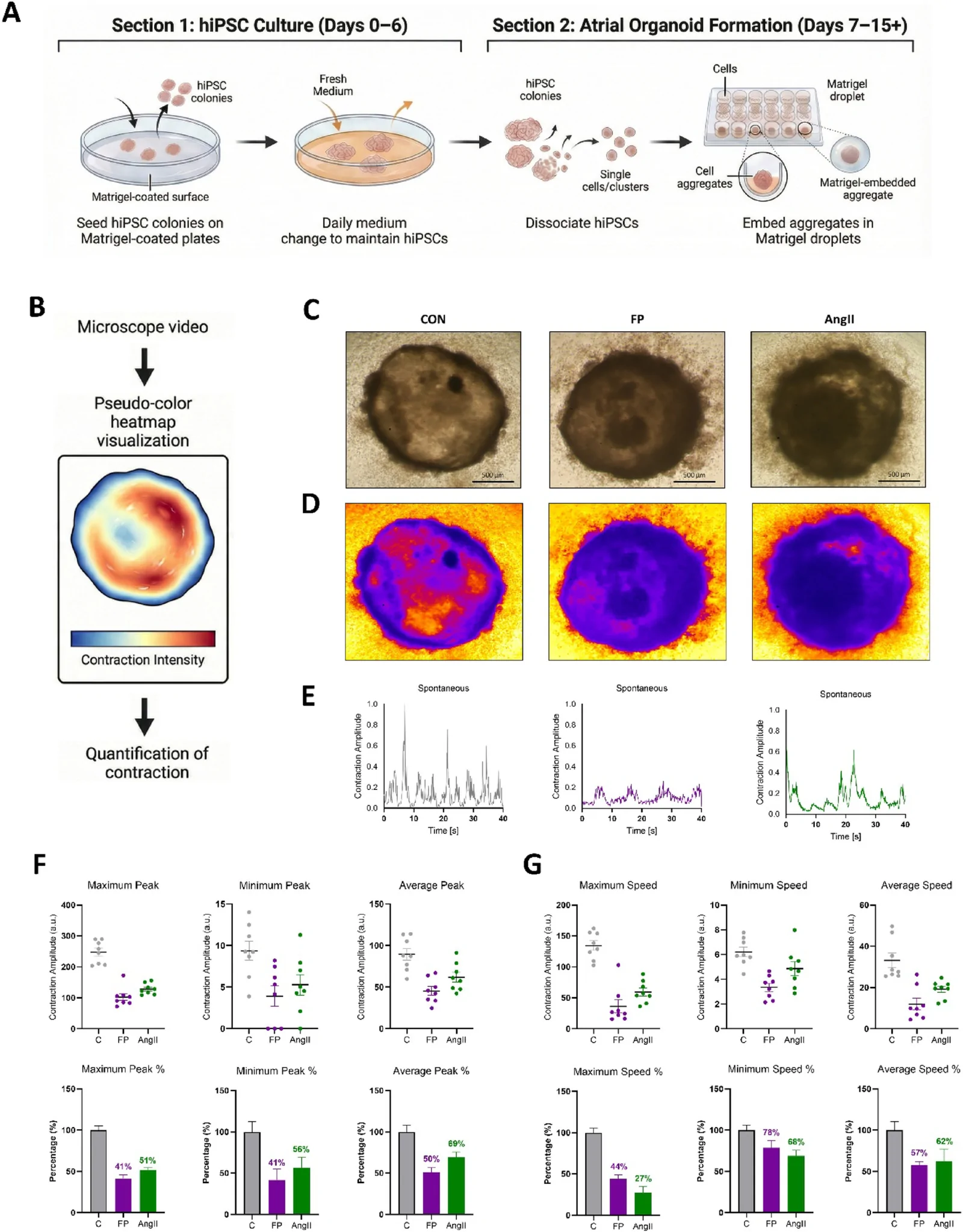
Contractile dysfunction in human atrial organoids induced by fast pacing and Angiotensin II. Functional assessment of human cardiac organoids exposed to atrial fibrillation (AF)-like conditions. **A** Schematic representation of the workflow for generating human atrial organoids from hiPSCs. “Background” section (Days 0–6): hiPSC culture. Human induced pluripotent stem cell (hiPSC) colonies were seeded onto Matrigel-coated plates and maintained under standard culture conditions. Medium was changed daily to preserve pluripotency and colony integrity. “Methods” section (Days 7–15 +): Atrial organoid formation. hiPSC colonies were dissociated into single cells or small clusters and allowed to form aggregates. Cell aggregates were subsequently embedded in Matrigel droplets to promote three-dimensional organization and organoid development. This process enables structural assembly and maturation toward atrial-like tissue. **B** Schematic contraction analysis workflow. Microscope videos of beating organoids were converted into pseudo-color heatmaps representing contraction intensity and subsequently quantified for contractile measurements. **C** Brightfield images show gross morphology of control (CON), fast-paced (FP), and Angiotensin II (AngII)-treated organoids. **D** Pseudocolor displacement heatmaps from MuscleMotion reveal contraction patterns; purple indicates minimal displacement, yellow/red reflects high activity. **E** Representative contraction traces highlight rhythmic peaks in CON and reduced, irregular patterns in FP and AngII groups. **F** Quantitative analysis of peak contraction amplitude, including maximum, minimum, and average peak height (top) and percent reduction (bottom). **G** Contraction speed metrics including maximum, minimum, and average contraction speed (top) with corresponding percentage reductions (bottom). Both FP and AngII organoids exhibit impaired contractility, with AngII displaying more pronounced suppression in some metrics. Data shown as mean ± SD. n ≥ 8 organoids/group

To model AF-associated mechanical dysfunction, organoids were subjected to electrical FP or AngII. Gross morphology remained unchanged across groups (Fig. 3A), but contractile performance was markedly impaired under both conditions. Displacement mapping with MuscleMotion showed bright, coordinated contraction zones in controls, whereas FP- and AngII-treated organoids exhibited darker, fragmented, and spatially restricted motion (Fig. 3B). Time-series traces confirmed rhythmic, high-amplitude contractions in controls, contrasted by low-amplitude oscillations in FP organoids and irregular, moderately reduced contractions in AngII organoids (Fig. 3C). Representative contraction videos illustrate these motion patterns and the corresponding color coding (Supplementary Video 1). Normalized to control values, fast pacing (FP) reduced maximum, minimum, and average peak amplitudes to 41%, 41%, and 50%, respectively, compared with 51%, 56%, and 69% under AngII treatment (Fig. 3D). In contrast, AngII more severely impaired contraction velocity, reducing the maximum speed to 27% of control, whereas FP reduced it to 44% (Fig. 3E). Minimum and average contraction speeds declined in both groups, with FP reducing them to 78% and 57%, and AngII to 68% and 62%, respectively. These findings indicate that FP primarily reduces contraction peak amplitude, while AngII affects contraction speed, highlighting distinct functional impacts.

### Mitochondrial gene suppression in AF datasets and organoid models

**Fig. 4:**
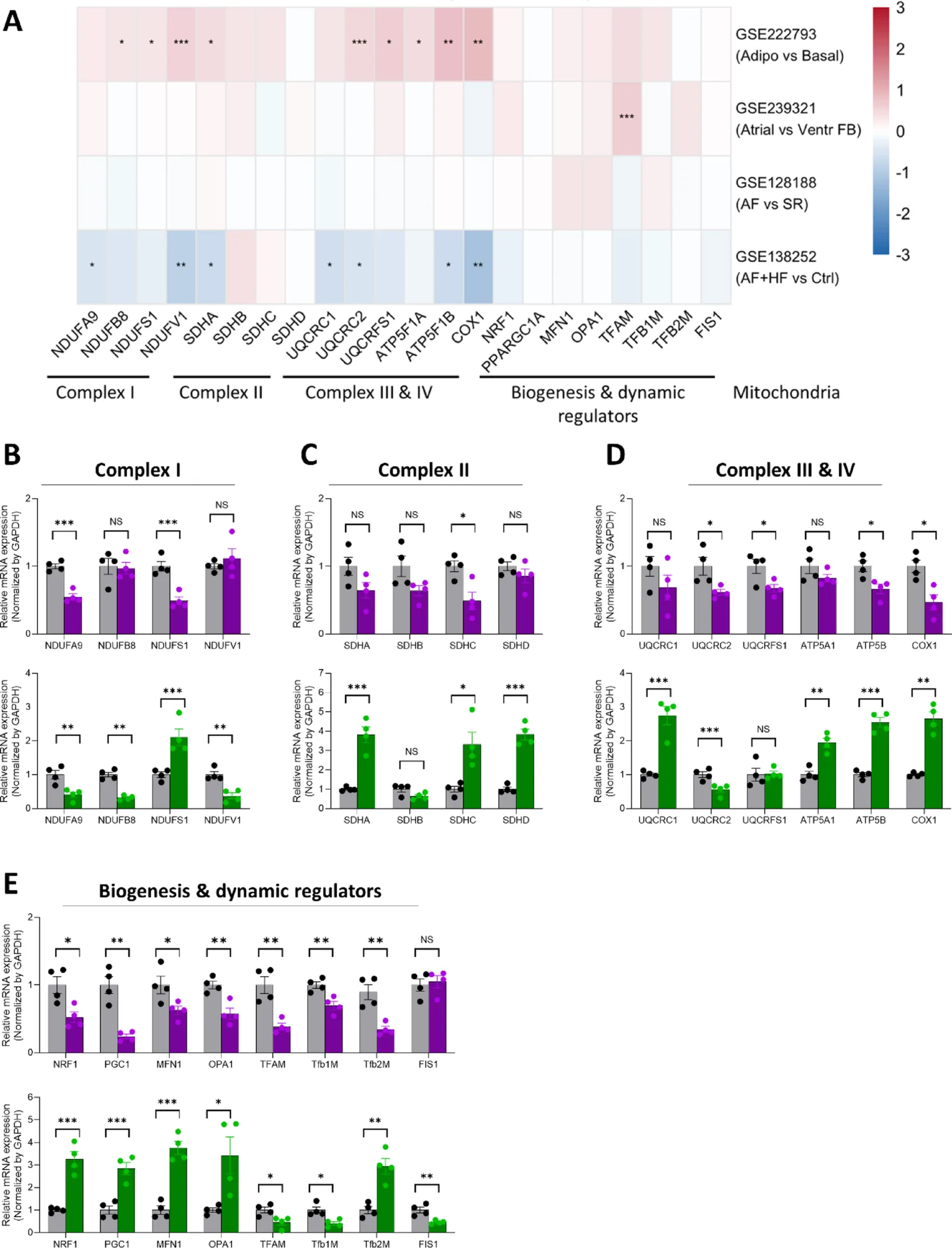
Transcriptomic and experimental validation of mitochondrial gene expression across the AF spectrum and organoid models. Mitochondrial subunit and regulator expression in AF GEO datasets and human atrial organoids. A Heatmap showing log₂ fold change of mitochondrial genes across four AF-related GEO datasets (GSE222793, GSE239321, GSE128188, and GSE138252). Genes include Complex I–IV subunits and mitochondrial biogenesis/dynamics regulators. Blue: downregulation; red: upregulation. (B–E) qPCR analysis of mitochondrial genes in FP (purple) and AngII-treated (green) human cardiac organoids, compared to control (gray). **B** Complex I genes (NDUFA9, NDUFB8, NDUFS1, NDUFV1), **C** Complex II genes (SDHA, SDHB, SDHC, SDHD), **D** Complex III/IV genes (UQCRC1, UQCRC2, UQCRFS1, ATP5A1, ATP5B, COX1), and **E** biogenesis and mitochondrial dynamic regulators (PGC1α, NRF1, MFN1, OPA1, TFAM, TFB1M, TFB2M, FIS1). All data are normalized to GAPDH and presented as mean ± SD. *p* < *0.05*, *p* < *0.01*, *p* < *0.001*

Analysis of 22 mitochondrial-related transcripts across four GEO datasets revealed variable expression patterns reflecting the biological model and comparison of each dataset (Fig. 4A). In GSE222793 (adipogenic vs basal), several genes showed upregulation, including Complex I subunits NDUFB8, NDUFS1, NDUFV1, and SDHA, Complex III subunits UQCRC1, UQCRC2, and UQCRFS1, and Complex V subunits ATP5F1A and ATP5F1B, while other transcripts remained largely unchanged. GSE239321 (atrial vs ventricular fibroblasts) showed broadly neutral expression across most mitochondrial genes, with the exception of TFAM which was significantly upregulated. GSE128188 (AF vs SR) showed modest and largely non-significant changes across mitochondrial transcripts. By contrast, GSE138252 (AF + HF vs control) displayed broad downregulation across most mitochondrial genes, including Complex I subunits NDUFA9 and NDUFV1, Complex II subunit SDHA, Complex III subunits UQCRC1 and UQCRC2, and Complex V subunit ATP5F1B, while remaining transcripts showed modest decreases or were unchanged. Together, these results indicate that broad mitochondrial suppression is a feature of AF with concurrent heart failure (GSE138252), while the other three datasets show heterogeneous and largely non-significant mitochondrial changes.

qPCR analysis of organoids confirmed distinct remodeling patterns between FP- and AngII-treated conditions (Fig. 4B–E). FP organoids showed widespread downregulation of ETC (Electron Transport Chain) subunits and biogenesis regulators, including NDUFA9 and NDUFS1 (Complex I), SDHC (Complex II), UQCRC2 and UQCRFS1 (Complex III), ATP5B (Complex V), and COX1 (Complex IV). Suppression was also observed in transcriptional and dynamic regulators NRF1, PGC1, MFN1, OPA1, TFAM, Tfb1M, and Tfb2M. In contrast, AngII organoids exhibited a mixed profile with both up- and downregulated subsets. Upregulated genes included NDUFS1 (Complex I); SDHA, SDHC, and SDHD (Complex II); UQCRC1 (Complex III); ATP5A1 and ATP5B (Complex V); COX1 (Complex IV); and the regulators NRF1, PGC1, MFN1, OPA1, and Tfb2M. Downregulated genes comprised NDUFA9, NDUFB8, and NDUFV1 (Complex I); SDHB (Complex II); UQCRC2 (Complex III); as well as TFAM, Tfb1M, and FIS. Together, these findings show that FP organoids reproduce a broad suppression of mitochondrial pathways, whereas AngII organoids display a more heterogeneous transcriptional profile with simultaneous increases and decreases across mitochondrial complexes.

### Mitochondrial protein expression differs between FP- and AngII-treated organoids

**Fig. 5:**
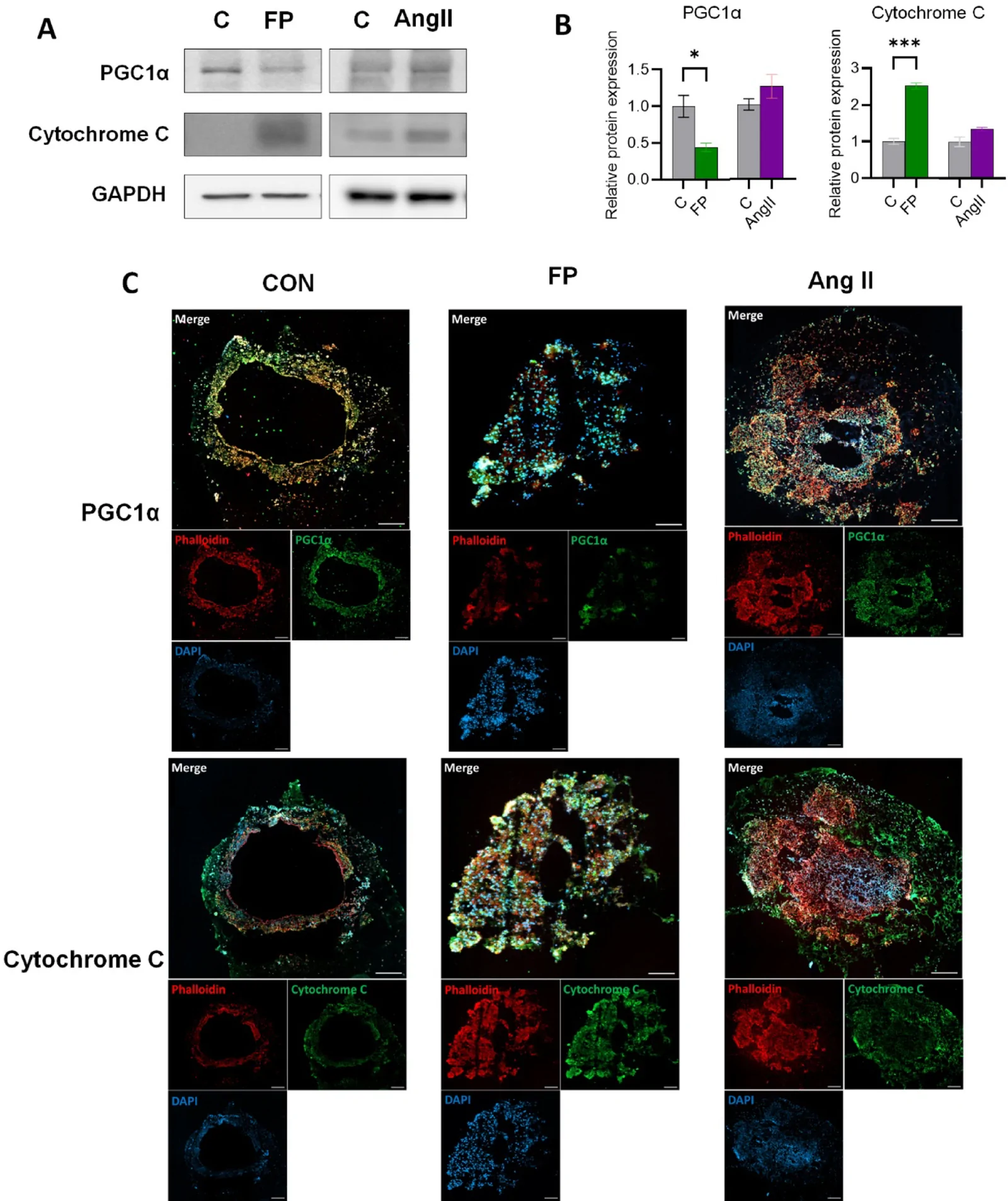
Protein-level suppression of mitochondrial markers in atrial organoids exposed to fast pacing or Angiotensin II. Mitochondrial protein expression is downregulated in human atrial organoids exposed to FP or AngII. A Western blot analysis of PGC1α and Cytochrome C in control, FP, and AngII-treated groups. GAPDH used as loading control. **B** Quantification of Western blot band intensity normalized to GAPDH. AngII-treated organoids show stronger suppression of both mitochondrial markers. **C** Immunofluorescent staining of organoid cross-sections. Top panels: co-staining for PGC1α (green), F-actin (Phalloidin, red), and nuclei (DAPI, blue). Bottom panels: co-staining for Cytochrome C (green), Phalloidin, and DAPI. Merged images show cytoskeletal disorganization and spatial loss of mitochondrial protein signals, particularly in AngII-exposed organoids. Scale bars = 100 μm

To examine whether transcriptional changes were reflected at the protein level, we assessed PGC1α and cytochrome C in FP- and AngII-treated organoids (Fig. 5A–B). FP organoids showed a marked reduction of PGC1α protein, while cytochrome C was clearly increased compared with controls. In contrast, AngII organoids displayed a slight induction of PGC1α together with a modest increase in cytochrome C. Immunofluorescence analysis confirmed these patterns (Fig. 5C). In controls, both proteins were broadly and uniformly distributed throughout the cytoplasm, aligned with organized F-actin. FP organoids exhibited reduced PGC1α signal and strong cytochrome C staining, whereas AngII organoids showed mild PGC1α induction and a weaker cytochrome C increase compared with FP.

These findings indicate that FP and AngII models exhibit distinct protein-level remodeling, with FP characterized by PGC1α suppression and strong cytochrome C induction, and AngII by modest induction of both proteins.

### FP and AngII organoids display distinct fibrotic remodeling signatures

**Fig. 6:**
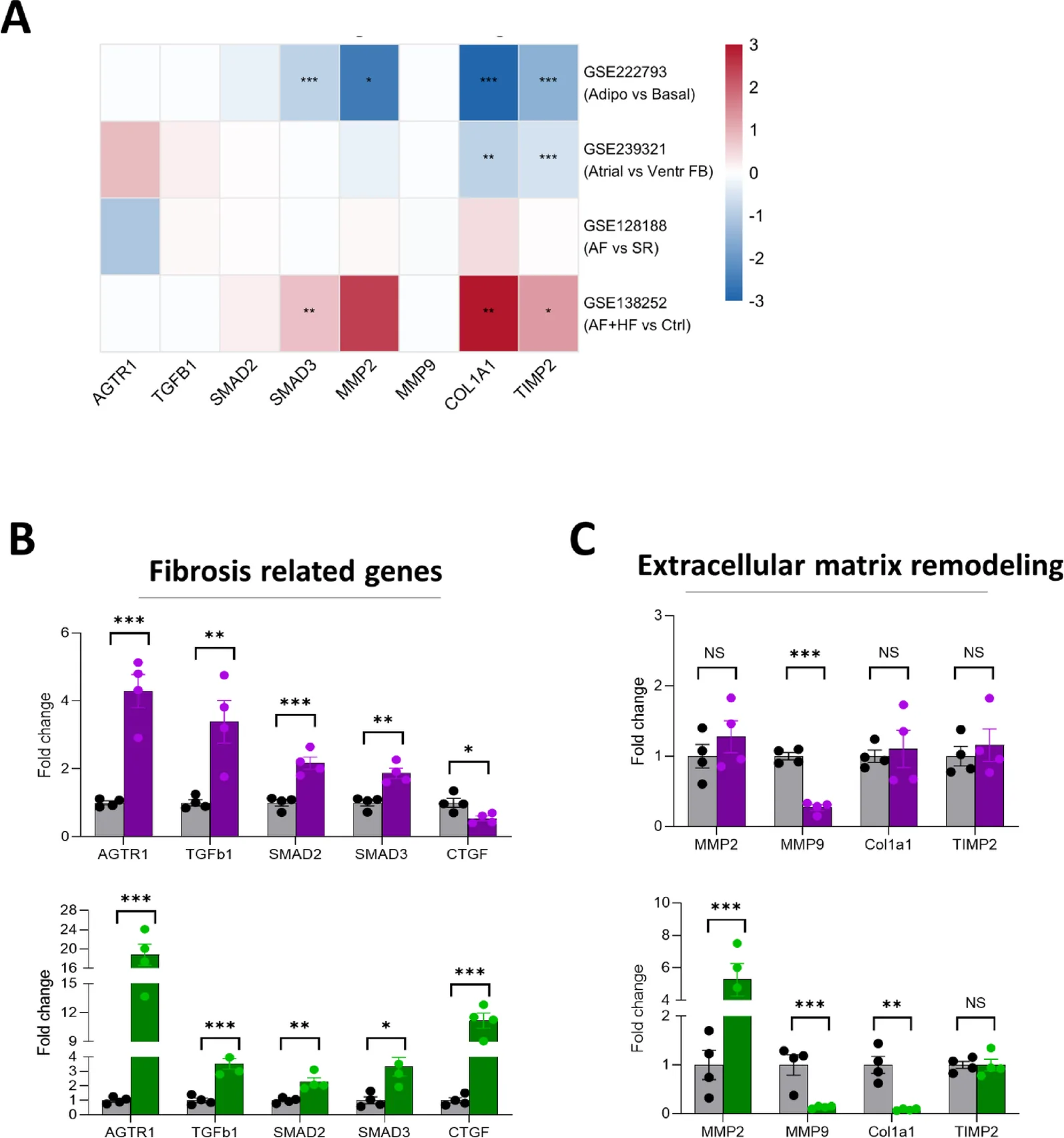
Fibrotic gene activation in human AF datasets and organoid models reflects divergent remodeling mechanisms. Upregulation of fibrosis-related genes in human atrial fibrillation (AF) datasets and organoid models. **A** Heatmap of fibrosis and ECM remodeling genes across GEO datasets (GSE222793, GSE239321, GSE128188, GSE138252), including AGTR1, TGFβ1, SMAD2/3, CTGF, MMP2/9, COL1A1, and TIMP2. Red: upregulated; blue: downregulated. **B** qPCR analysis of fibrosis pathway genes in fast-paced (FP, purple) and Angiotensin II (AngII, green) organoids, showing increased expression of AGTR1, TGFβ1, SMAD2/3, and CTGF. **C** ECM remodeling genes (MMP2, MMP9, COL1A1, TIMP2) are significantly upregulated in AngII-treated organoids compared to controls, with milder effects in FP. Data shown as mean ± SD; *p* < *0.05*, *p* < *0.01*, *p* < *0.001*

**Fig. 7:**
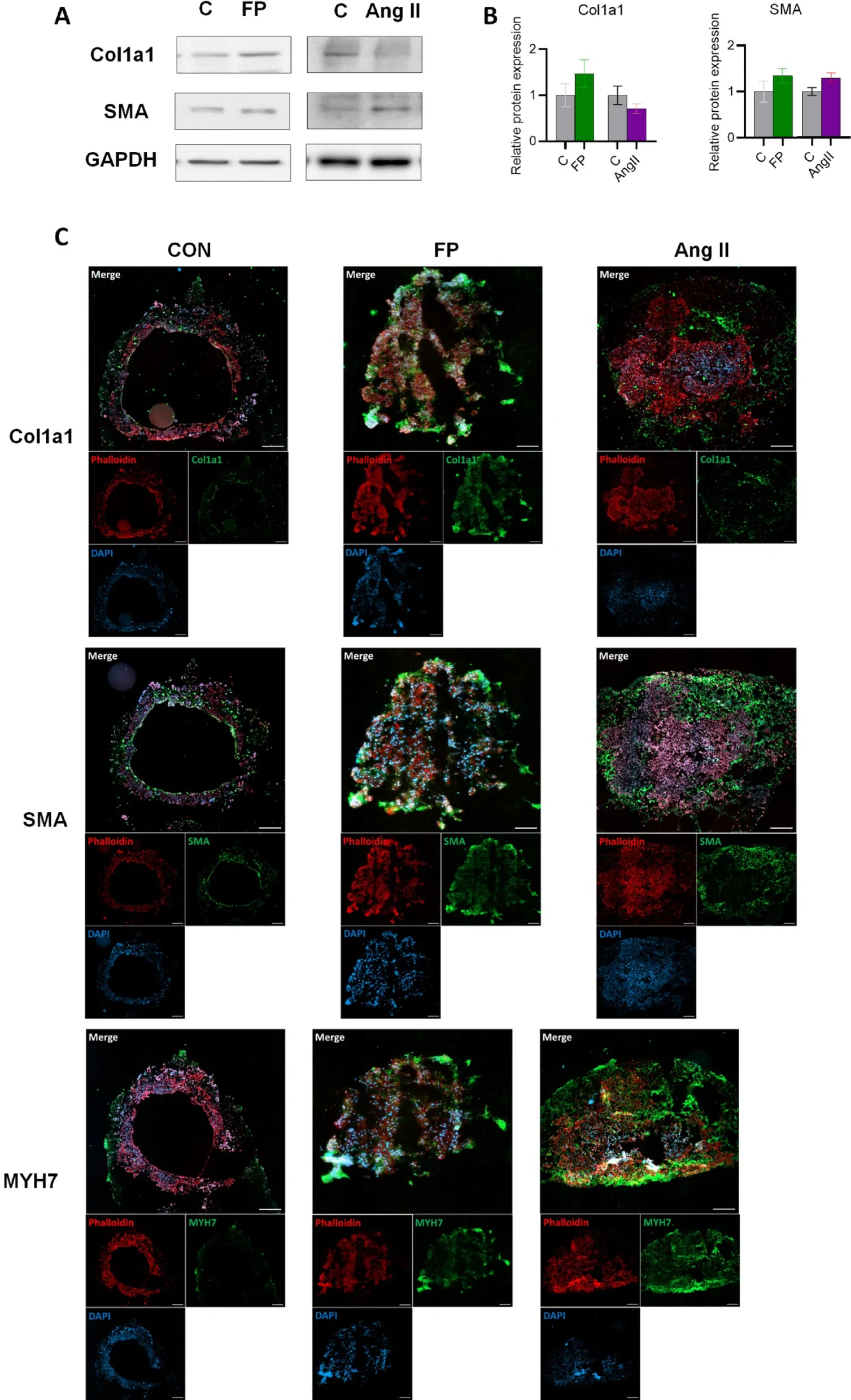
Fibrotic protein expression and tissue remodeling in atrial organoids under FP and AngII stress. Validation of fibrosis in atrial organoids using fibrotic protein markers. **A** Western blot analysis of Col1a1 and αSMA in control (C), FP, and AngII-treated organoids. GAPDH used as loading control. **B** Quantification of band intensities normalized to GAPDH. Both FP and AngII treatments increase Col1a1 and SMA protein expression, with AngII inducing a stronger response. **C** Immunofluorescent staining of organoid cross-sections for Col1a1 (top) and SMA (bottom), co-labeled with Phalloidin (F-actin, red) and DAPI (nuclei, blue). Merged images reveal widespread matrix remodeling and myofibroblast marker expression in FP and AngII groups. AngII-treated organoids exhibit diffuse, dense fibrosis consistent with contractile impairment. Scale bars = 100 μm

GSE222793 exhibited significant downregulation of ECM remodeling genes including SMAD3, MMP2, COL1A1, and TIMP2. GSE239321 showed downregulation of COL1A1 and TIMP2, while GSE128188 showed largely neutral expression across fibrosis-associated transcripts. By contrast, GSE138252 displayed significant upregulation of SMAD3, MMP2, COL1A1, and TIMP2, indicating active fibrotic remodeling in advanced AF with concurrent heart failure (Fig. 6A). In cardiac organoids, FP and AngII induced distinct transcriptional profiles (Fig. 6B–C). FP organoids upregulated AGTR1, TGFβ1, SMAD2, and SMAD3, while CTGF expression was reduced. ECM remodeling genes showed minimal change under FP, except for a decrease in MMP9. In contrast, AngII organoids upregulated all fibrosis-related genes, including AGTR1, TGFβ1, SMAD2, SMAD3, and CTGF, with ECM remodeling characterized by induction of MMP2, reduction of MMP9 and COL1A1, and no change in TIMP2. At the protein level, FP and AngII again demonstrated distinct patterns (Fig. 7A–B). FP organoids showed increased COL1A1 and a modest rise in αSMA, whereas AngII organoids exhibited reduced COL1A1 with a slight increase in αSMA. Immunofluorescence analysis confirmed these observations (Fig. 7C). Control organoids showed minimal staining, FP organoids displayed stronger COL1A1 and αSMA signals, and AngII organoids exhibited weaker COL1A1 with modest αSMA staining. The COL1A1 signal in AngII samples appeared higher by immunostaining than by Western blot. Taken together, these findings suggest that FP is associated with activation of TGFβ/SMAD signaling and increased COL1A1 expression, whereas AngII is associated with broader profibrotic gene upregulation but reduced COL1A1 levels and modest αSMA induction.

### Targeted concordance analysis demonstrates that FP and AngII organoids model distinct contexts of the AF remodeling spectrum

**Fig. 8:**
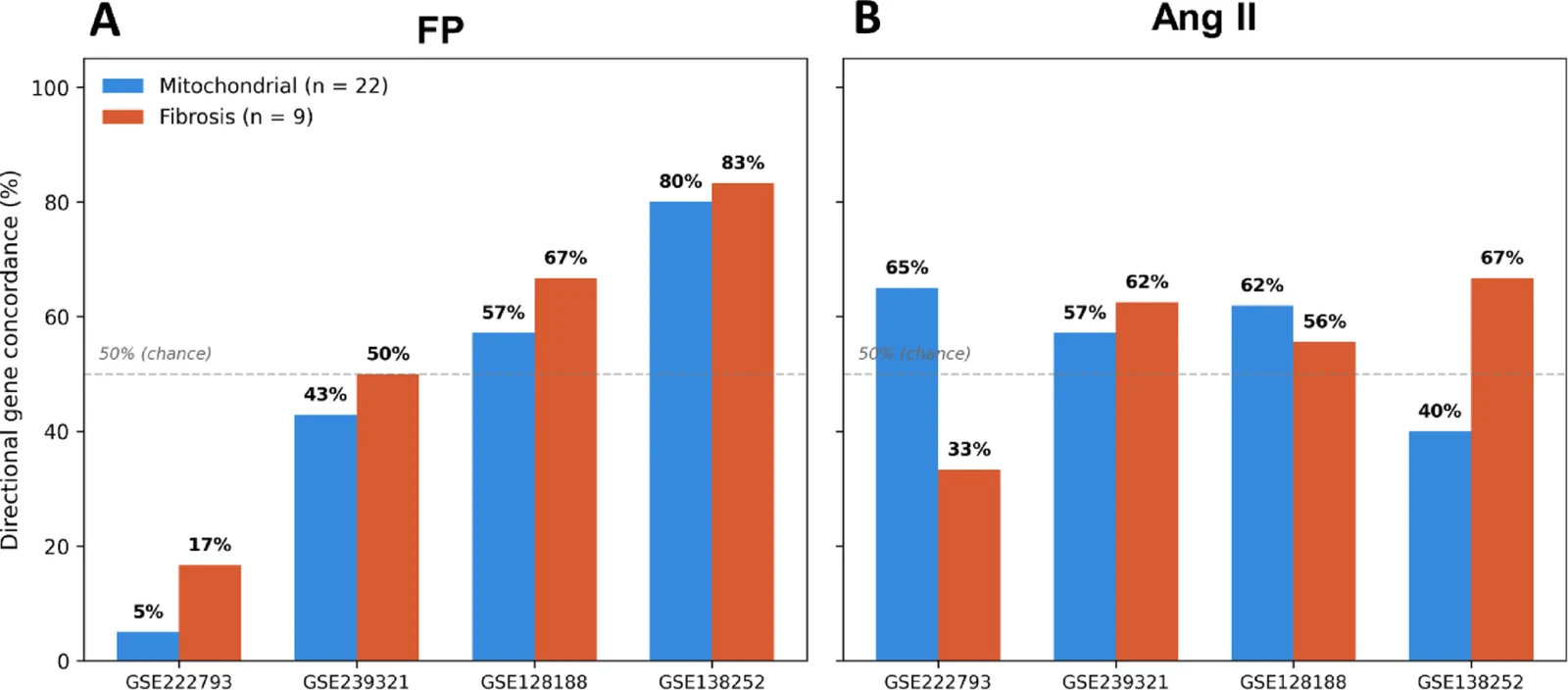
Targeted directional concordance between atrial organoid remodeling models and AF-spectrum transcriptomic datasets. Quantitative comparison of FP and AngII organoid responses with four GEO datasets representing complementary AF-spectrum contexts. **A** Directional concordance of FP organoids against each GEO dataset, calculated separately for mitochondrial (n = 22, blue) and fibrosis-related (n = 9, orange) gene panels. FP shows the highest concordance with GSE138252 (AF with heart failure), with 80% mitochondrial and 83% fibrosis concordance. **B** Directional concordance of AngII organoids using the same approach. AngII shows the highest mitochondrial concordance with GSE222793 (cardiometabolic atrial remodeling; 65%) and the highest fibrosis concordance with GSE138252 (67%). Dashed horizontal line indicates the 50% reference level (chance)

To provide a quantitative comparison between experimentally induced organoid remodeling and the AF-spectrum transcriptomic datasets, we performed a targeted directional concordance analysis using the mitochondrial and fibrosis-related genes measured by qPCR in the organoids (Fig. 8). Because genome-wide transcriptomic profiling was not performed in the present study, the analysis was restricted to genes directly evaluated by qPCR and compared with their corresponding log₂ fold-change direction in each GEO dataset. FP organoids exhibited the highest directional concordance with GSE138252 (AF with heart failure), indicating greater similarity of the experimentally measured mitochondrial and fibrosis-related gene programs to this disease context than to the other GEO datasets (80% mitochondrial concordance, 83% fibrosis concordance). In contrast, AngII organoids exhibited the highest mitochondrial concordance with GSE222793 (cardiometabolic atrial remodeling; 65%) and the highest fibrosis concordance with GSE138252 (67%). These findings suggest that AngII reproduces molecular features associated with cardiometabolic mitochondrial remodeling while concurrently inducing profibrotic remodeling. Among the four AF-spectrum datasets, FP exhibited the greatest directional concordance with the AF with heart failure dataset (GSE138252), whereas AngII showed greater concordance with the cardiometabolic remodeling dataset (GSE222793) than with GSE138252. Collectively, these targeted concordance analyses demonstrate that FP and AngII do not represent identical models of atrial fibrillation, but instead reproduce distinct molecular remodeling programs within the broader atrial fibrillation spectrum.

## Discussion

This study investigated AF by examining gene expression patterns of mitochondrial dysfunction and fibrosis identified in publicly available datasets and testing whether these features could be reproduced in human atrial organoids. Two well-known in vitro inducers of AF, FP to simulate electrophysiological overload and AngII to model neurohormonal activation [43–47], were applied to human atrial organoids to examine their structureal and molecular remodeling responses. FP was associated with reduced contractile function, downregulation of mitochondrial transcripts, and mild fibrotic changes, partially resembling patterns reported in AF with heart failure. AngII treatment caused contractile dysfunction accompanied by variable mitochondrial changes and upregulation of profibrotic genes, resembling profiles more typical of AF without heart failure. Importantly, aberrant AngII signaling is a central feature of the neurohormonal dysregulation observed in cardiometabolic diseases, including diabetes and obesity. Therefore, the divergent signatures elicited by AngII in our model may also offer insights into the specific profibrotic and metabolic remodeling trajectories that characterize AF in patients with co-existing metabolic syndrome or diabetic cardiomyopathy [48–50]. These findings suggest that FP and AngII may influence atrial remodeling through distinct molecular patterns. The observed transcriptomic similarities indicate that FP aligns more closely with AF accompanied by heart failure, whereas AngII aligns with AF alone. Further studies using larger and more diverse datasets will be necessary to determine which model more accurately represents specific AF phenotypes.

Although mitochondrial dysfunction was known to be a key downregulated signature in AF [51–53], the downregulated enrichment heatmap showed undetectable mitochondrial pathways. This likely reflects the biological heterogeneity inherent to the four GEO datasets, which were selected to represent complementary stages of the AF spectrum, from cardiometabolic predisposition and structural substrate formation to established chronic AF and advanced AF with heart failure. This intentional heterogeneity was reflected in the pathway level analysis, where mitochondrial gene sets did not show a consistent enrichment signal across all datasets. Mitochondrial suppression was most pronounced in GSE138252, consistent with bioenergetic failure in advanced AF with concurrent heart failure, while the remaining datasets showed variable or opposing mitochondrial enrichment reflecting distinct disease stages and cellular contexts. At the gene level, however, core ETC subunits showed more consistent directional changes across AF-relevant datasets, suggesting that mitochondrial dysfunction is a genuine feature of AF remodeling that is partially obscured at the pathway level by biological heterogeneity across the AF spectrum. In contrast, fibrosis-associated pathways showed the highest statistical significance across datasets by Fisher combined meta-analysis. However, the direction of enrichment differed across datasets, reflecting the distinct biological context of each model rather than a uniform fibrotic signature. The partial separation observed in PCA across datasets further underscores the biological heterogeneity inherent to multi cohort transcriptomic integration, highlighting the importance of interpreting pathway level enrichment in the context of dataset specific biological characteristics rather than as a uniform disease signature.

The AF + HF dataset (GSE138252) showed uniform and profound mitochondrial downregulation, whereas the three other datasets showed variable responses, with some mitochondrial genes reduced and others remaining stable or elevated. These transcriptomic patterns were also observed in our organoid models, where FP reproduced the pronounced mitochondrial suppression typical of AF + HF, while AngII represented the fibrotic remodeling pattern seen in AF-only cohorts.

The targeted directional concordance analysis further supports that FP and AngII represent distinct experimental models within the atrial fibrillation remodeling spectrum. FP demonstrated the greatest molecular agreement with the AF with heart failure transcriptomic dataset (GSE138252), consistent with its pronounced mitochondrial suppression and profibrotic remodeling. In contrast, AngII showed greater similarity to the cardiometabolic remodeling dataset (GSE222793) for mitochondrial genes while maintaining substantial concordance with fibrosis-associated signatures. Importantly, these analyses were intentionally restricted to experimentally measured mitochondrial and fibrosis-related genes and therefore provide a targeted molecular concordance analysis rather than whole transcriptome equivalence. Together, these findings reinforce the concept that FP and AngII model complementary aspects of AF-associated remodeling rather than a single uniform disease phenotype.

A key strength of the organoid platform is its multicellular composition. Unlike single-lineage cardiomyocyte cultures, Cardiac organoids integrate multiple cell types that support paracrine signaling, fibroblast myocyte interactions, and extracellular matrix deposition, collectively recapitulating tissue level processes that contribute to AF progression. This multicellular complexity is particularly valuable for investigating the interaction between mitochondrial dysfunction and fibrosis, as mitochondrial stress induces oxidative damage and profibrotic signaling, whereas matrix accumulation amplifies mitochondrial dysfunction by increasing tissue stiffness and restricting metabolic exchange. [2, 54, 55]. Although organoids cannot fully replicate the architecture or systemic context of human atria, they provide a useful intermediate system between 2D cultures and animal models, particularly when aligned with human transcriptomic data.

From a clinical perspective, these findings reinforce that AF is not solely an electrical arrhythmia but also a chronic tissue disorder characterized by persistent metabolic and structural remodeling. Even after restoration of sinus rhythm, fibrosis and energetic compromise may persist, contributing to recurrence and functional decline. Therapies that stabilize mitochondrial function or attenuate fibrotic remodeling may therefore complement current rhythm-control and anticoagulation strategies and address unmet needs in AF management.

## Limitations

Several limitations should be acknowledged. The experimental paradigms used in this study, including FP and AngII stimulation, are simplified representations of in vivo stressors. Their dosage, duration, and kinetics may not accurately reflect the variable exposures experienced by patients. FP does not account for the fluctuating burden of atrial tachyarrhythmia in clinical settings, and acute AngII treatment captures only one component of chronic renin–angiotensin–aldosterone system activation. Moreover, organoid models lack key physiological elements such as vascularization, immune cell populations, and mechanical load, all of which play important roles in atrial remodeling in vivo. In addition, quantitative characterization of cellular composition, including fibroblast to cardiomyocyte ratios and dynamic changes in cell populations during remodeling, was beyond the scope of the present study. Consequently, the fibrotic responses reported here should be interpreted as remodeling associated molecular and protein signatures rather than direct measurements of fibroblast abundance. The transcriptomic datasets analyzed for comparison are heterogeneous in origin and clinical context, including both surgical tissues and stem cell derived in vitro models, which complicates direct translation. Furthermore, several individual GEO cohorts included relatively small sample sizes, which may limit statistical power. Although this limitation was partially mitigated by integrating four complementary AF-spectrum datasets and performing targeted directional concordance analysis, validation in larger patient cohorts will further strengthen the generalizability of these findings. Despite these limitations, the repeated identification of mitochondrial and fibrotic remodeling across organoid models and human data supports the robustness and biological relevance of these findings.

## Conclusion

In summary, this study demonstrates that electrophysiological and neurohormonal stressors drive distinct but overlapping remodeling trajectories in atrial organoids, characterized by mitochondrial dysfunction and fibrosis. While these models cannot fully reproduce the complexity of human AF, they provide a tractable platform for mechanistic investigation and for generating hypotheses on stage-specific therapeutic strategies that extend beyond rhythm control.

## Supplementary Information

Below is the link to the electronic supplementary material.

Supplementary Material 1

Supplementary Material 2

Supplementary Material 3

Supplementary Material 4

Supplementary Material 5

Supplementary Material 6

Supplementary Material 7

Supplementary Material 8

Supplementary Material 9

Supplementary Material 10

## Data Availability

Data will be made available on request.

## Abbreviations

AF: Atrial fibrillation
AngII: Angiotensin II
GEO: Gene expression omnibus
hiPSCs: Human-induced pluripotent stem cells
PBS: Phosphate buffered saline
FP: Rapid pacing
ROS: Reactive oxygen species
