# Supplementary material for: Divergent mitochondrial and fibrotic signatures in atrial fibrillation: insights from organoids and GEO data
Source: Cardiovasc Diabetol. 2026 Jul 11;25:287. doi: 10.1186/s12933-026-03291-0 (PMC13602660; doi:10.1186/s12933-026-03291-0)
Supplement: Supplementary file 1 [file 12933_2026_3291_MOESM10_ESM.docx]

**Divergent mitochondrial and fibrotic signatures in atrial fibrillation: Insights from organoids and GEO data**

Jihee Won^a^, Hyocheol Jung^b^, Jin Chul Kim^b^, Ji-Eun Jeong^b^, Young Il Park^b^ Biaggio Uricoli^a^, Sanghee Lee^c^, Young Hoon Son^b,^* and Gun‑Jae Jeong^c,d^*

^a^ Coulter Department of Biomedical Engineering, Georgia Institute of Technology & Emory University School of Medicine, Atlanta, GA 30322, USA.

^b^ Center for Specialty Chemicals, Division of Specialty and Bio-based Chemical Technology, Korea Research Institute of Chemical Technology, Ulsan 44412, Republic of Korea

^c^ School of Nanomedical Engineering, Korea National University of Transportation, Chungju 27469, Republic of Korea

^d^ Institute of Cell and Tissue Engineering, College of Medicine, The Catholic University of Korea, Seoul 06591, Republic of Korea

*Corresponding authors

*E-mail address*: young.hoon.son@emory.edu (Y.H. Son) and gunjae.jeong@ut.ac.kr(G.J. Jeong)

**Supplementary Table 1.** Human primer sequences used for real-time quantitative reverse transcriptase polymerase chain reaction (qRT-PCR)

| **Gene** | **Forward** | **Reverse** |
| --- | --- | --- |
| NDUFA9 | GCCCATTTGAGCCCTGGATAAC | GCCTTGAGTTCCAGTGGTGTTG |
| NDUFB8 | CCACACCTGTTTCTTGGCATGTC | ATCACCGCCTCGTTCCAGGTAC |
| NDUFS1 | GAGTGGACTCTGACACCTTATGC | ACAACATCTGCCTCTTCCACACC |
| NDUFV1 | TGTGTGAGACGGTGCTGATGGA | CGATGGCTTTCACGATGTCCGT |
| SDHA | GAGATGTGGTGTCTCGGTCCAT | GCTGTCTCTGAAATGCCAGGCA |
| SDHB | GCAGTCCATAGAAGAGCGTGAG | TGTCTCCGTTCCACCAGTAGCT |
| SDHC | GGTTCAAACCGTCCTCTGTCTC | CGACATGCCAAAAAGAGAGACCC |
| SDHD | GCAGCACATACACTTGTCACCG | GGGAATAGTCCATCGCAGAGCA |
| UQCRC1 | TGTCTCGTGCAGACTTGACCGA | GCGAGGTCTAACAGTTGCTGGT |
| UQCRC2 | CCGTGGAATTGAAGCAGTTGGTG | CTGTGGTGACATTGAGCAGGAAC |
| UQCRFS1 | CCTGTGTTGGACCTGAAGCGG | CAGAGAAGTCAGGCACCTTGATG |
| COX1(PTGS1) | GATGAGCAGCTTTTCCAGACGAC | AACTGGACACCGAACAGCAGCT |
| ATP5A1 | GCTCCTTACTCTGGCTGTTCCA | GCGGAGCAACAGAGACATCTGA |
| ATP5B (ATP5F1B) | TCATGCTGAGGCTCCAGAGTTC | ACAGTCTTGCCAACTCCAGCAC |
| PGC1a | CCAAAGGATGCGCTCTCGTTCA | CGGTGTCTGTAGTGGCTTGACT |
| NRF1 | GGCAACAGTAGCCACATTGGCT | GTCGTCTGGATGGTCATCTCAC |
| TFAM | GTGGTTTTCATCTGTCTTGGCAAG | TTCCCTCCAACGCTGGGCAATT |
| Tfb1M | AGCAGCCATTCAAGCTGGTGGA | GTCTGCCAACTCTAACAGCCTG |
| Tfb2M | GGGAAAACCAAGTAGACCTCCAC | TTTCGAGCGCAACCACTTTGGC |
| OPA1 | GTGGTTGGAGATCAGAGTGCTG | GAGGACCTTCACTCAGAGTCAC |
| MFN1 | GGTGAATGAGCGGCTTTCCAAG | TCCTCCACCAAGAAATGCAGGC |
| FIS1 | CAAGGAACTGGAGCGGCTCATT | GGACACAGCAAGTCCGATGAGT |
| AGTR1 | CAGCGTCAGTTTCAACCTGTACG | GCAGGTGACTTTGGCTACAAGC |
| TGF-β1 | TACCTGAACCCGTGTTGCTCTC | GTTGCTGAGGTATCGCCAGGAA |
| MMP2 | AGCGAGTGGATGCCGCCTTTAA | CATTCCAGGCATCTGCGATGAG |
| MMP9 | GCCACTACTGTGCCTTTGAGTC | CCCTCAGAGAATCGCCAGTACT |
| TIMP2 | ACCCTCTGTGACTTCATCGTGC | GGAGATGTAGCACGGGATCATG |
| CTGF (CCN2) | CTTGCGAAGCTGACCTGGAAGA | CCGTCGGTACATACTCCACAGA |
| COL1A1 | GATTCCCTGGACCTAAAGGTGC | AGCCTCTCCATCTTTGCCAGCA |
| Smad2 | GGGTTTTGAAGCCGTCTATCAGC | CCAACCACTGTAGAGGTCCATTC |
| Smad3 | TGAGGCTGTCTACCAGTTGACC | GTGAGGACCTTGTCAAGCCACT |

**Supplementary Video 1. Beating of cardiac organoids under control, FP, and AngII conditions**

Representative videos show contraction patterns of human cardiac organoids under control, FP, and AngII stimulation. Control organoids exhibit bright, coordinated, and rhythmic contractions. FP organoids display reduced and fragmented motion, while AngII organoids show irregular and spatially restricted contractions.

**Supplementary Data 1.** Differentially expressed genes in GSE128188 (AF vs SR, human left atrial appendage tissue) identified by limma-voom analysis. Includes gene symbol, log₂ fold change, p-value, Benjamini-Hochberg adjusted p-value, and moderated t-statistic.

**Supplementary Data 2.** Differentially expressed genes in GSE138252 (AF+HF vs NF, human left atrial cardiomyocytes) identified by limma-voom analysis. Includes gene symbol, log₂ fold change, p-value, Benjamini-Hochberg adjusted p-value, and moderated t-statistic.

**Supplementary Data 3.** Differentially expressed genes in GSE222793 (adipogenic vs basal medium, iPSC-derived atrial-like cardiomyocytes) identified by limma-voom analysis. Includes gene symbol, log₂ fold change, p-value, Benjamini-Hochberg adjusted p-value, and moderated t-statistic.

**Supplementary Data 4.** Differentially expressed genes in GSE239321 (atrial vs ventricular fibroblasts, primary human cardiac fibroblasts) identified by limma-voom analysis. Includes gene symbol, log₂ fold change, p-value, Benjamini-Hochberg adjusted p-value, and moderated t-statistic.

**Supplementary Data 5.** Gene Set Enrichment Analysis (GSEA) results across all four GEO datasets. GSEA was performed in GSEAPreranked mode against MSigDB Hallmark and GO Biological Process collections. Includes pathway name, normalized enrichment score (NES), nominal p-value, FDR q-value, and leading edge genes for each dataset.

**Supplementary Data 6.** Cross-dataset pathway convergence analysis using Fisher's combined probability method. Includes pathway name, per-dataset FDR values, combined p-value, and combined FDR across the four GEO datasets.
